# Supplementary material for: Treatment of Metastatic or High-Risk Solid Cancer Patients by Targeting the Immune System and/or Tumor Burden: Six Cases Reports
Source: Int J Mol Sci. 2019 Nov 28;20(23):5986. doi: 10.3390/ijms20235986 (PMC6929121; doi:10.3390/ijms20235986)
Supplement: Supplementary file 1 [file ijms-20-05986-s001.pdf]

Suppl. Table 1a. Other findings from immunological assessment in patient 1 receiving inhibiting immune-suppression therapy.

**Patient 1**									
Parameter	Time	D	Mean	SD	p-value*	Median	Minimum	Maximum	p-value**
CD8(n)	Basal	2	476.0	33.9	0.354	476.0	452.0	500.0	0.248
	IT	3	534.0	66.9		508.0	484.0	610.0	
NK(n)	Basal	2	215.0	15.6	0.380	215.0	204.0	226.0	0.374
	IT	3	184.0	39.0		177.0	149.0	226.0	
Treg(n)	Basal	2	19.0	5.7	0.329	19.0	15.0	23.0	0.083
	IT	3	25.6	2.0		25.0	24.0	27.9	
CD4+45R0(n)	Basal	2	364.5	44.5	0.222	364.5	333.0	396.0	0.083
	IT	3	519.3	131.5		493.0	403.0	662.0	
IL1(pg/ml)	Basal	2	1.2	0.9	0.448	1.2	0.5	1.8	0.374
	IT	3	1.9	0.3		1.8	1.7	2.2	
IL1R(pg/ml)	Basal	2	330.0	14.1	0.024	330.0	320.0	340.0	0.083
	IT	3	464.3	41.2		478.0	418.0	497.0	
IL6(pg/ml)	Basal	2	8.0	6.5	0.433	8.0	3.4	12.6	0.248
	IT	3	2.4	1.7		2.7	0.6	4.0	
IL8(pg/ml)	Basal	2	1.8	1.6	0.234	1.8	0.6	2.9	0.083
	IT	3	5.2	2.9		3.8	3.3	8.6	
IL10(pg/ml)	Basal	2	2.2	0.1	0.096	2.2	2.1	2.3	0.083
	IT	3	2.6	0.2		2.6	2.4	2.8	
IL2R(ng/ml)	Basal	2	2.8	0.1	0.395	2.8	2.7	2.9	0.197
	IT	3	2.7	0.1		2.7	2.6	2.7	
TNFalpha(pg/ml)	Basal	2	5.8	4.0	0.681	5.8	3.0	8.6	1.000
	IT	3	4.3	0.9		4.7	3.2	4.9	
TGFB1(pg/ml)	Basal	2	67.5	19.1	0.689	67.5	54.0	81.0	1.000
	IT	3	60.3	2.5		60.0	58.0	63.0	
FGF(pg/ml)	Basal	2	8.2	3.0	0.538	8.2	6.1	10.3	0.564
	IT	3	6.7	2.1		6.6	4.6	8.8	
EGF(pg/ml)	Basal	2	21.5	2.1	0.028	21.5	20.0	23.0	0.083
	IT	3	11.3	3.1		12.0	8.0	14.0	
IGFI(ng/ml)	Basal	2	121.5	0.7	0.585	121.5	121.0	122.0	0.564
	IT	3	120.3	2.5		120.0	118.0	123.0	
PDFG(pg/ml)	Basal	2	1066.5	429.2	0.341	1066.5	763.0	1370.0	0.083
	IT	3	577.3	143.3		655.0	412.0	665.0	
MCP-1(pg/ml)	Basal	2	250.5	33.2	0.058	250.5	227.0	274.0	0.083
	IT	3	467.7	94.7		444.0	387.0	572.0	

D: determination; IT: inhibiting immune-suppression therapy; Ly tot: total lymphocytes; CD8. CD4+25+(Treg). CD4+45R0 (memory cells): monoclonal antibody T subsets; NK: CD16+56+. natural killer cells; IL: interleukin; IL1R: interleukin 1 receptor; IL2R: interleukin 2 receptor; TNFalpha: tumor necrosis factor alpha; TGFB1:tumor growth factor beta1; FGF: fibroblast growth factor; EGF: epidermal growth factor; IGFI: insulin-like growth factor-1; PDFG: platelet-derived growth factor; MCP-1: monocyte chemo-attractant protein-1; *t-test; **Mann-Whitney test

Suppl. Table 1b. Other findings from immunological assessment in patient 2 receiving inhibiting immune-suppression therapy.

**Patient 2**									
Parameter	Time	D	Mean	SD	p-value*	Median	Minimum	Maximum	p-value**
Ly tot(n)	Basal	2	1141.0	54.0	0.576	1141.5	1103.0	1180.0	0.564
	IT	3	1174.0	59.0		1146.0	1135.0	1242.0	
CD4(n)	Basal	2	639.0	29.7	0.297	639.0	618.0	660.0	0.564
	IT	3	660.3	8.7		658.0	653.0	670.0	
CD8(n)	Basal	2	171.0	8.5	0.818	171.0	165.0	177.0	0.564
	IT	3	176.7	29.7		160.0	159.0	211.0	
CD3(n)	Basal	2	856.0	41.0	0.498	856.0	827.0	885.0	0.564
	IT	3	884.5	40.3		863.0	859.5	931.0	
NK(n)	Basal	2	165.5	16.3	0.728	165.5	154.0	177.0	0.564
	IT	3	160.5	13.3		160.0	147.5	174.0	
Treg (n)	Basal	2	36.0	2.8	0.011	36.0	34.0	38.0	0.076
	IT	3	25.0	1.7		26.0	23.0	26.0	
CD4+45R0(n)	Basal	2	478.5	6.4	0.773	478.5	474.0	483.0	1.000
	IT	3	466.3	51.6		481.0	409.0	509.0	
CD19(n)	Basal	2	119.5	2.1	0.742	119.5	118.0	121.0	0.564
	IT	3	117.7	6.7		116.0	112.0	125.0	
IL1(pg/ml)	Basal	2	3.8	3.2	0.359	3.8	1.5	6.0	0.083
	IT	3	0.2	0.3		116.0	112.0	125.0	
IL1R(pg/ml)	Basal	2	355.5	47.4	0.032	355.5	322.0	389.0	0.083
	IT	3	200.3	43.4		181.0	170.0	250.0	
IL6(pg/ml)	Basal	2	7.5	7.0	0.824	7.5	2.5	12.4	1.000
	IT	3	6.0	2.2		6.4	3.7	8.0	
IL8(pg/ml)	Basal	2	1.0	0.1	0.008	1.0	0.9	1.0	0.076
	IT	3	0.1	0.2		0.0	0.0	0.3	
IL10(pg/ml)	Basal	2	1.7	0.5	0.911	1.7	1.3	2.0	0.564
	IT	3	1.8	1.2		1.1	1.0	3.2	
IL2R(ng/ml)	Basal	2	3.5	0.2	0.584	3.5	3.3	3.6	0.564
	IT	3	3.7	0.5		3.7	3.2	4.1	
TNFalpha(pg/ml)	Basal	2	7.0	0.3	0.321	7.0	6.8	7.2	0.083
	IT	3	12.9	6.6		9.6	8.5	20.5	
TGFB1(pg/ml)	Basal	2	63.1	0.1	0.962	63.1	63.0	63.2	0.564
	IT	3	61.6	40.2		40.0	36.7	108.0	
FGF(pg/ml)	Basal	2	2.2	0.8	0.485	63.1	63.0	63.2	0.248
	IT	3	1.4	1.2		1.2	0.3	2.7	
EGF(pg/ml)	Basal	2	21.0	12.7	0.459	21.0	12.0	30.0	0.564
	IT	3	45.3	37.5		45.0	8.0	83.0	
IGFI(ng/ml)	Basal	2	164.5	10.6	0.030	164.5	157.0	172.0	0.083
	IT	3	196.0	7.8		192.0	191.0	205.0	
PDFG(pg/ml)	Basal	2	5607.5	2527.9	0.901	5607.5	3820.0	7395.0	1.000
	IT	3	5903.3	2324.6		5495.0	3810.0	8405.0	
VEGF(pg/ml)	Basal	2	207.5	70.0	0.638	207.5	158.0	257.0	0.564
	IT	3	164.7	98.6		152.0	73.0	269.0	
MCP-1(pg/ml)	Basal	2	516.5	67.2	0.573	516.5	469.0	564.0	0.554
	IT	3	468.3	90.6		416.0	416.0	573.0	

D: determination; IT: inhibiting immune-suppression therapy; Ly tot: total lymphocytes; CD3. CD4.CD8.CD4+25+ (Treg). CD4+45R0 (memory cells): monoclonal antibody T subsets; NK: CD16+56+. natural killer cells; CD19: monoclonal antibody B lymphocytes; IL: interleukin; IL1R: interleukin 1 receptor; IL2R: interleukin 2 receptor; TNFalpha: tumor necrosis factor alpha; TGFB1: tumor growth factor beta1; FGF: fibroblast growth factor; EGF: epidermal growth factor; IGFI: insulin-like growth factor-1; PDFG: platelet-derived growth factor; VEGF: vascular endothelial growth; MCP-1:monocyte

chemo-attractant protein-1 factor; * t-test; **Mann-Whitney test

Suppl. Table 1c. Other findings from immunological assessment in patient 3 receiving inhibiting immune-suppression therapy.

**Patient 3**									
Parameter	Time	D	Mean	SD	p-value*	Median	Minimum	Maximum	p-value**
Ly tot(n)	Basale	2	1380.0	17.0	0.984	1380.0	1368.0	1392.0	0.564
	IT	3	1383.0	188.0		1428.0	1177.0	1544.0	
CD8(n)	Basale	2	282.5	6.4	0.723	282.5	278.0	287.0	0.564
	IT	3	271.7	37.0		271.0	235.0	309.0	
CD3(n)	Basale	2	958.5	2.1	0.354	958.5	957.0	960.0	0.139
	IT	3	907.0	63.2		928.0	836.0	957.0	
NK(n)	Basale	2	110.0	1.4	0.612	110.0	109.0	111.0	0.564
	IT	3	126.3	38.8		143.0	82.0	154.0	
Treg(n)	Basale	2	34.3	0.1	0.759	34.3	34.2	34.3	0.564
	IT	3	35.0	3.0		35.0	32.0	38.0	
CD4+45R0	Basale	2	345.0	4.2	0.299	345.0	342.0	348.0	0.083
	IT	3	459.3	122.4		401.0	377.0	600.0	
CD19(n)	Basale	2	303.5	3.5	0.530	303.5	301.0	306.0	0.564
	IT	3	349.3	86.8		357.0	259.0	432.0	
IL1(pg/ml)	Basale	2	0.2	0.2	0.112	0.2	0.0	0.3	0.083
	IT	3	0.8	0.4		0.9	0.4	1.1	
IL1R(pg/ml)	Basale	2	284.5	318.9	0.593	284.5	59.0	510.0	1.000
	IT	3	451.7	47.5		450.0	405.0	500.0	
IL6(pg/ml)	Basale	2	1.3	0.1	0.538	1.3	1.2	1.4	0.564
	IT	3	1.7	0.8		2.0	0.8	2.4	
IL8(pg/ml)	Basale	2	0.6	0.6	0.374	0.6	0.2	1.0	0.374
	IT	3	0.2	0.3		0.2	0.0	0.5	
IL10(pg/ml)	Basale	2	1.9	0.5	0.619	1.9	1.5	2.2	0.564
	IT	3	2.2	0.9		2.4	1.3	3.0	
IL12(pg/ml)	Basale	2	132.0	21.2	0.758	132.0	117.0	147.0	1.000
	IT	3	126.0	6.2		124.0	121.0	133.0	
IL2R(ng/ml)	Basale	2	1.6	0.1	0.550	1.6	1.5	1.7	0.361
	IT	3	1.7	0.2		1.8	1.5	1.8	
TNFalpha(pg/ml)	Basale	2	4.9	2.7	0.901	4.9	3.0	6.8	1.000
	IT	3	4.6	0.7		5.0	3.8	5.0	
TGFB1(pg/ml)	Basale	2	21.8	1.9	0.079	21.8	20.4	23.1	0.083
	IT	3	39.7	9.1		41.1	30.0	48.0	
FGF(pg/ml)	Basale	2	3.1	0.0	0.038	3.1	3.1	3.1	0.076
	IT	3	3.8	0.3		3.7	3.6	4.1	
EGF(pg/ml)	Basale	2	4.0	4.2	0.272	4.0	1.0	7.0	0.197
	IT	3	10.0	5.2		7.0	7.0	16.0	
IGFI(ng/ml)	Basale	2	202.5	12.0	0.061	202.5	194.0	211.0	0.083
	IT	3	166.3	14.2		174.0	150.0	175.0	
PDFG(pg/ml)	Basale	2	880.5	391.0	0.718	880.5	604.0	1157.0	0.564
	IT	3	1064.7	558.7		820.0	670.0	1704.0	
MCP-1(pg/ml)	Basale	2	399.5	12.0	0.345	399.5	391.0	408.0	0.564
	IT	3	333.3	78.9		311.0	268.0	421.0	
D: determination; IT: inhibiting immune-suppression therapy; Ly tot: total lymphocytes; CD3. CD8. CD4+25+(Treg) . CD4+45R0 (memory cells): monoclonal antibody T subsets; NK: CD16+56+. natural killer cells; CD19: monoclonal antibody B lymphocytes; IL: interleukin; IL1R: interleukin 1 receptor; IL2R: interleukin 2 receptor; TNFalpha: tumor necrosis factor alpha; TGFB1: tumor growth factor beta1; FGF: fibroblast growth factor; EGF: epidermal growth factor; IGFI: insulin-like growth factor-1; PDFG: platelet-derived growth factor; MCP-1: monocyte chemo-attractant protein-1; * t-test; **Mann-Whitney									

test

Suppl. Table 1d. Other findings from immunological assessment in patient 4 receiving inhibiting immune-suppression therapy.

**Patient 4**									
Parameter	Time	D	Mean	SD	p-value*	Median	Minimum	Maximum	p-value**
CD4(n)	Basale	3	641.0	39.9	0.190	661.0	595.0	667.0	0.127
	IT	3	573.7	62.1		555.0	523.0	643.0	
NK(n)	Basale	3	330.3	109.7	0.324	345.0	214.0	432.0	0.275
	IT	3	249.7	58.2		266.0	185.0	298.0	
TREG(n)	Basale	3	33.3	13.3	0.708	30.0	22.0	48.0	0.827
	IT	3	28.8	14.1		33.5	13.0	40.0	
CD4+45R0(n)	Basale	3	350.3	126.2	0.212	374.0	214.0	463.0	0.275
	IT	3	235.3	45.6		247.0	185.0	274.0	
IL1(pg/ml)	Basale	3	1.0	0.6	0.300	0.7	0.6	1.7	0.050
	IT	3	5.6	5.8		2.8	1.8	12.3	
IL1R(pg/ml)	Basale	3	171.3	65.7	0.217	157.0	114.0	243.0	0.275
	IT	3	112.7	22.2		125.0	87.0	126.0	
IL6(pg/ml)	Basale	3	11.1	12.8	0.396	4.0	3.4	25.9	0.513
	IT	3	19.0	2.5		20.3	16.1	20.7	
IL8(pg/ml)	Basale	3	3.1	0.9	0.898	3.2	2.2	4.0	0.507
	IT	3	2.7	4.7		0.0	0.0	8.2	
IL10(pg/ml)	Basale	3	5.4	7.5	0.196	1.6	0.6	14.0	0.275
	IT	3	18.7	12.9		12.0	10.6	33.6	
IL12(pg/ml)	Basale	3	81.0	39.8	0.155	58.0	58.0	127.0	0.178
	IT	3	127.0	22.0		127.0	105.0	149.0	
IL2R(ng/ml)	Basale	3	3.4	2.9	0.123	1.8	1.7	6.7	0.077
	IT	3	7.5	0.8		7.6	6.7	8.2	
TNFalpha(pg/ml)	Basale	3	6.1	4.3	0.662	4.4	2.9	11.0	0.513
	IT	3	7.5	2.6		7.1	5.1	10.2	
TGFB1(pg/ml)	Basale	3	34.9	6.8	0.506	36.0	27.6	41.0	0.658
	IT	3	41.3	13.8		36.0	31.0	57.0	
FGF(pg/ml)	Basale	3	1.8	1.2	0.308	2.2	0.4	2.8	0.513
	IT	3	2.8	0.7		2.7	2.1	3.5	
EGF(pg/ml)	Basale	3	7.7	5.5	0.707	5.0	4.0	14.0	0.658
	IT	3	6.3	1.5		6.0	5.0	8.0	
IGFI(pg/ml)	Basale	3	117.3	14.2	0.101	125.0	101.0	126.0	0.050
	IT	3	139.0	10.5		138.0	129.0	150.0	
PDFG(pg/ml)	Basale	3	618.0	277.4	0.578	492.0	426.0	936.0	0.827
	IT	3	939.3	877.6		498.0	370.0	1950.0	
VEGF(pg/ml)	Basale	3	77.7	30.7	0.201	74.0	49.0	110.0	0.275
	IT	3	49.0	10.8		52.0	37.0	58.0	
MCP-1(pg/ml)	Basale	3	379.3	63.6	0.044	400.0	308.0	430.0	0.050
	IT	3	193.3	91.5		159.0	124.0	297.0	

D: determination; IT: inhibiting immune-suppression therapy; Ly tot: total lymphocytes; CD4. CD4+25+(Treg) . CD4+45R0 (memory cells): monoclonal antibody T subsets; NK: CD16+56+. natural killer cells; IL: interleukin; IL1R: interleukin 1 receptor; IL2R: interleukin 2 receptor; TNFalpha: tumor necrosis factor alpha; TGFB1: tumor growth factor beta1; FGF: fibroblast growth factor; EGF: epidermal growth factor; IGFI: insulin-like growth factor-1; PDFG: platelet-derived growth factor; VEGF: vascular endothelial growth factor;MCP-1: monocyte chemo-attractant protein-1; * t-test; **Mann-Whitney test
